# Bioactive Compounds and Antioxidant Activity of Fresh and Processed White Cauliflower

**DOI:** 10.1155/2013/367819

**Published:** 2013-09-22

**Authors:** Fouad A. Ahmed, Rehab F. M. Ali

**Affiliations:** Department of Biochemistry, Faculty of Agriculture, Cairo University, Giza 12613, Egypt

## Abstract

*Brassica* species are very rich in health-promoting phytochemicals, including phenolic compounds, vitamin C, and minerals. The objective of this study was to investigate the effect of different blanching (i.e., water and steam) and cooking (i.e., water boiling, steam boiling, microwaving, and stir-frying) methods on the nutrient components, phytochemical contents (i.e., polyphenols, carotenoids, flavonoid, and ascorbic acid), antioxidant activity measured by DPPH assay, and phenolic profiles of white cauliflower. Results showed that water boiling and water blanching processes had a great effect on the nutrient components and caused significant losses of dry matter, protein, and mineral and phytochemical contents. However, steam treatments (blanching and cooking), stir-frying, and microwaving presented the lowest reductions. Methanolic extract of fresh cauliflower had significantly the highest antioxidant activity (68.91%) followed by the extracts of steam-blanched, steam-boiled, stir-fried, and microwaved cauliflower 61.83%, 59.15%, 58.93%, and 58.24%, respectively. HPLC analysis revealed that the predominant phenolics of raw cauliflower were protocatechuic acid (192.45), quercetin (202.4), pyrogallol (18.9), vanillic acid (11.90), coumaric acid (6.94), and kaempferol (25.91) mg/100 g DW, respectively.

## 1. Introduction

Plant-based foods such as fruit, vegetables, and whole grains, which contain significant amounts of bioactive phytochemicals, may provide desirable health benefits beyond basic nutrition to reduce the risk of chronic diseases. Epidemiological evidence suggests that consumption of a diet rich in vegetables and fruits has positive implications for human health [1]. In the last decades, special attention has been paid towards edible plants, especially those that are rich in secondary metabolites (frequently called phytochemicals), and nowadays, there is an increasing interest in the antioxidant activity of such phytochemicals present in diet. Recent reports suggest that cruciferous vegetables act as a good source of natural antioxidants due to the high levels of carotenoids, tocopherols, and ascorbic acid, and strong epidemiological evidence shows that these compounds may help to protect the human body against damage by reactive oxygen species. In addition to carotenoids, tocopherols, and ascorbic acid, most of the antioxidative effect related to plant food intake is mainly due to the presence of phenolic compounds, which have been associated with flavour and colour characteristics of fruits and vegetables. In this aspect, the popularity and consumption of vegetable *Brassica* species is increasing because of their nutritional value. *Brassica* crops have been related to the reduction of the risk of chronic diseases including cardiovascular diseases and cancer. *Brassica* foods are very nutritive, providing nutrients and health-promoting phytochemicals such as vitamins, carotenoids, fiber, soluble sugars, minerals, glucosinolates, and phenolic compounds [2]. Vegetables of Brassicaceae family are the essential sources of phenolic compounds in the human diet. They also contain derivatives of hydroxycinnamic, caffeic, chlorogenic, ferulic, and synapic acids as well as flavonols (kaempferol derivatives, and quercetin derivatives) and anthocyanins (red cabbage) [3, 4] *Brassica* vegetables have been reported as good sources of antimutagens. Several epidemiological studies showed that they were associated with reduction of cancer [5]. Further studies of experimental animals also demonstrated that feeding some of these vegetables could inhibit the development of some chemically induced carcinogenesis [6] and resulted in the induction of phase 2 detoxifying enzymes such as glutathione S-transferase. It has been reported that natural compounds in these vegetables were effective in protection against chemical carcinogenesis by modulating carcinogen metabolism [7]. Cauliflower, like broccoli and cabbage, belongs to the cruciferous (Brassicaceae) family of vegetables, which has been shown to be effective in fighting certain forms of cancer. Cauliflower is so closely related to broccoli that both are designated as the same variety of the cruciferous family, which not only share the wonderful phytochemicals, but also contain nutritive value of vitamin A, thiamine, riboflavin, niacin, vitamin C, calcium, iron, phosphorous, and fat to help fight diseases [8]. Cauliflower is an important vegetable grown all over the world and has a wide variety of uses directly as a vegetable or as an ingredient in salads, soups, and so forth. Cauliflower occupies an area of 8.88 million ha, having a production of 16.40 million tons in the world [9]. The aim of the current investigation was to determine the effect of different blanching and cooking methods on the nutritional quality of white cauliflower by assessing the proximate composition, the count of total polyphenols, total flavonoids, total carotenoids and vitamin C, and fractionation of phenolic compounds using HPLC as well as antioxidant activity by DPPH of raw and processed cauliflower.

## 2. Material and Methods

### 2.1. Cauliflower

Freshly harvested cauliflowers (*Brassica oleracea var*. *botrytis* L.) were purchased from the local market on the day of processing (Giza Governorate, Egypt) during the winter season (December 2012). Cauliflower heads were selected at marketable stage, free from insect or mechanical damage. Cauliflower heads were transported to the laboratory of biochemistry department, Faculty of Agriculture, Cairo University, Egypt, where the inedible parts were removed using a sharp knife. The cauliflower was cut into almost equal small bite-sized pieces (edible florets, about 5 cm), mixed well, and divided into portions (200 g). 

### 2.2. Pretreatments 

#### 2.2.1. Water Blanching

Approximately 1000 mL of water was poured into a stainless steel vessel and heated at 100°C. Cauliflower florets (200 g) were immersed in the boiling water at 100°C for 3 min. The samples were drained on a stainless sieve until cold and then weighed. 

#### 2.2.2. Steam Blanching

Steam blanching was conducted by suspending 200 g of cauliflower florets above 1000 mL of boiling water for 3 min in a stainless steel steamer with a lid. The samples were drained on a stainless sieve until cold and then weighed.

#### 2.2.3. Cooking Treatments

The time taken for the completeness of cooking was determined after three initial standardization trials. For all cooking treatments, the minimum cooking time to reach a similar tenderness for an adequate palatability and taste, according to the Egyptian eating habits, was used.

#### 2.2.4. Water Boiling

Approximately 1000 mL of water was poured into a stainless steel vessel, which was then covered with a lid and heated to boiling. Cauliflower florets (200 g) were added to the boiling water and cooked for 6 min. The samples were drained on a stainless sieve until cold and then weighed. 

#### 2.2.5. Steam Boiling

Steam boiling was conducted by suspending 200 g of cauliflower florets above 1000 mL of boiling water for 6 min 15 s. in a stainless steel steamer with a lid. The samples were drained on a stainless sieve until cold and then weighed.

#### 2.2.6. Microwave Cooking

A microwave oven (Olympic Electric, Model Kor-1AOA, 2450 MHz, Korea) at full power (1000 W) was used for microwaving. Cauliflower florets (200 g) were placed in a glass beaker (Type Birex, England), and 10 mL water was added to prevent cauliflower from being burned during cooking and then cooked in a microwave oven for 3 min 30 s. The samples were drained on a stainless sieve until cold and then weighed.

*Stir-Frying*. Cauliflower florets (200 g) were placed in heated (140 ± 2°C) sunflower oil (10 mL) and stir-fried for 4 min 30 s. At the end of each trial, samples were drained off and dabbed with blotting paper to allow for the absorption of exceeding oil. 

Fresh and processed samples were kept at −25°C until analysis.

### 2.3. Analytical Methods

#### 2.3.1. Chemical Composition

Moisture, crude oil, crude protein (*N* × 6.25), and total ash of samples were determined as described in [10]. The minerals, that is, Ca, Fe, Zn, K, Cu, Me, Mg, and Na, were determined in a dilute solution of the ashed samples by atomic absorption spectrophotometer (3300 PerkinElmer) on described in [10]. The results are expressed as dry weight (DW) basis. 

#### 2.3.2. Ascorbic Acid Determination

Ascorbic acid content was determined according to [11]. Official Method 985.33 (2, 6-dichloroindophenol titrimetric method). Ascorbic acid content is expressed as mg/100 g DW.

#### 2.3.3. Total Carotenoids Determination

Total carotenoids were determined according to the procedure given by [12] as follows: 5 g of cauliflower florets were grinded and extracted with a mixture of acetone and petroleum ether (1 : 1, v/v) repeatedly using the mortar and pestle until a colorless residue was obtained. The upper phase was collected and combined with crude extracts after being washed for several times with water. The extracts were made up to a known volume with petroleum ether. Total carotenoids content was determined by recording the absorbance at 451 nm with a spectrophotometer. Total carotenoids were estimated by mg/100 g DW.

#### 2.3.4. Determination of Total Polyphenols

Total phenolics were determined using the Folin-Ciocalteu reagent [13]. Samples (2 g) were homogenized in 80% aqueous ethanol at room temperature and centrifuged in cold at 10 000 rpm for 15 min at 4°C, and the supernatant was preserved. The residue was reextracted twice with 80% ethanol, and supernatants were pooled and evaporated to dryness at room temperature. Residue was dissolved in 5 mL of distilled water. One hundred microliter of this extract was diluted to 3 mL with water and 0.5 mL of Folin-Ciocalteu reagent was added. After 3 min, 2 mL of 20% of sodium carbonate was added, and the contents were mixed thoroughly. After standing for 60 min at room temperature, the absorbance was measured at 650 nm. Phenolic contents were calculated on the basis of the standard curve for gallic acid (GAL). The results were expressed as mg of gallic acid equivalent per 100 g DW. 

#### 2.3.5. Determination of Total Flavonoids

The total flavonoids content was determined using the Dowd method [14]. 5 mL of 2% aluminium trichloride (AlCl_3_) in methanol was mixed with the same volume of the methanolic extract solution (0.4 mg/mL). After ten minutes the absorbance was measured at 415 nm using PerkinElmer UV-VIS Lambda.Blank sample consisting of a 5 mL extract solution with 5 mL methanol without AlCl_3_. The total flavonoid content was determined using a standard curve with catechin (0–100 mg/L) as the standard. Total flavonoids content is expressed as mg of catechin equivalents (CE)/100 g DW.

#### 2.3.6. Measurement of Antioxidant Activity by DPPH

*Extraction*. Ten g (10 g) of raw and processed cauliflower was homogenized with 100 mL methanol for 1 min and centrifuged at 10 000 rpm for 15 min at 4°C. The clear supernatant was transferred to a glass bottle and measured immediately for total antioxidant activity using DPPH assay. 100 *μ*L of sample was added to 5 mL DPPH solution, and the absorbance of DPPH reagent was determined at 515 nm after 30 min of incubation [15]. The inhibition percentage of the absorbance was calculated as follows:

(1) $$\begin{array}{c} \text{Inhibition}\%=\frac{\left(\text{Abs}\;t_{0}-\text{Abs}\;t_{30}\;\min\right)}{\text{Abs}\;t_{0}\times 100}. \end{array}$$

Abs *t*_0_ min was the absorbance of DPPH at time 0. Abs *t*_30_ min was the absorbance of DPPH after 30 min of incubation.

#### 2.3.7. Analysis of Phenolic Compounds Using HPLC

Phenolic compounds of methanolic extract of raw and processed cauliflower were identified using a method introduced by [16]. Briefly, HPLC analyses were performed using an HP1100 system with a thermostatically controlled column oven and a UV detector was set at 280 nm (Hewlett-Packard, Palo Alto, CA, USA). Samples and mobile phases were filtrated through a 0.45 mm Millipore filter, type GV (Millipore, Bedford, MA, USA), prior to HPLC injection. Each sample was analyzed in triplicate. The identified phenolic compounds were quantified on the basis of their peak area and compared with calibration curves obtained with the corresponding standards and then expressed as mg/100 g of extract.

#### 2.3.8. Statistical Analysis

Data were statistically analyzed in completely randomized design in factorial arrangement, according to the procedures outlined by [17], and the treatments means were compared by least significant differences (LSD) and Duncan' multiple range using SPSS program package. 

## 3. Results and Discussion

### 3.1. Chemical Composition

Chemical compositions of raw, blanched, and cooked cauliflower are presented in Table 1. Blanching and boiling processes caused significant increases in moisture content; percent increments ranged from 1.4% to 4.33%. This increase in moisture content might be due to leaching of water-soluble nutrients during blanching and boiling processes [18]. The highest (*P* < 0.05) loss of dry matter was observed for water-boiled cauliflower. During boiling, cellulose is little affected, but the middle lamella gets broken down by heat, thus making vegetables to take up water as the starch gelatinizes. The longer the boiling time, therefore, the higher the moisture content [19]. However, stir-fried cauliflower had significantly (*P* ≤ 0.05) lower moisture contents than raw, blanched, boiled, and microwaved cauliflowers. During the frying process, water evaporates from inside the product being fried, creating voids that are penetrated by the oil. As long as there is moisture to evaporate, the product will stay at a temperature of roughly 100°C [20]. As the water evaporates, the crust indicative of fried foods is formed on the exterior of the product. Fresh cauliflower has significantly the higher level of protein 27.77%. Blanching, boiling, microwaving, and stir-frying caused significant reductions in crude protein; these reductions were related to the protein denaturation at high temperature [21] during blanching and cooking processes. It is possible that soluble proteins were lost by leaching to the surrounding water [12]. The highest loss in protein content was observed for stir-fried cauliflower. This reduction was related to the fact that during the high temperature of stir-frying, both protein and soluble sugar participated in Maillard reaction and then resulted in their content decline [12]. The lowest loss was observed for microwaved cauliflower (3.16%). Studies on the effect of microwaves on the nutritional values of food proteins and amino acid content of peas and potatoes have concluded that microwave treatments do not have any significant effect on proteins [22]. Stir-frying process caused significant increase in fat content by 7.52% as dry weight basis. The absorption of fat through frying caused an increase of dry matter [23, 24], whereas water boiling and blanching caused significant decrease in fat content; these decreases were related to fat oxidation at high temperature [21] and could also be due to leaching effect [25]. No significant changes in fiber content were observed after blanching, steam boiling, microwaving, and stir-frying treatments of cauliflower. However, significant increase in fiber content was observed after water boiling of cauliflower. This increase may be attributed to the significant loss of dry matter into the boiling water [26]. Some studies have also suggested that heat processing can change the solubility and other physicochemical properties of fiber [27]. However, most studies analyzing crude and dietary fiber reported no significant changes in crude or dietary fiber after canning and freezing [28]. Fresh cauliflower had significantly the highest levels of ash 10.57%. The minerals leached from the cauliflower florets into the water at different rates during blanching, cooking treatments. Blanching, boiling, microwaving and stir-frying treatments significantly (*P* ≤ 0.05) decreased the ash contents of cauliflower florets. The highest (*P* ≤ 0.05) reduction in the ash content was observed for water-boiled cauliflower. Souci et al. [29] reported a loss of ash in boiled as compared with raw carrots. These decreases might be attributed to their diffusion into cooking water, while steam treatments (blanching and cooking), stir-frying, and microwaving gave the lowest reduction in the ash content. Losses of minerals during cooking are not caused by destruction but only by leaching into the cooking water [30]. No significant (*P* ≥ 0.05) changes in carbohydrate content were observed after blanching, boiling, and microwaving treatments of cauliflower. However, a significant (*P* ≤ 0.05) decrease in carbohydrate content was observed after stir-frying of cauliflower. This decrease may be attributed to the significant increase of fat content during frying process. 

### 3.2. Minerals

Mineral contents of raw, blanched, and cooked cauliflower are presented in Table 2. The highest (*P* ≤ 0.05) reduction in mineral content was observed for water-boiled cauliflower. The minerals leached from cauliflower florets into the water during water boiling process. However, steam blanching, steam boiling, microwaving, and stir-frying treatments resulted in the greatest retention of all minerals. Steam blanching is usually expected to conserve more soluble nutrients than water blanching [31]. Mineral losses in deep fried foods vary from 1% in potatoes to 26% in beef, being significantly lower than in boiled foods of the same type [32]. Boiling leads to high losses while stewing, steaming, microwave cooking, and even pressure steaming cause only small losses [33]. Losses of minerals during cooking are not caused by destruction but only by leaching into the cooking water [34]. Water blanching and boiling resulted in the greatest reduction of potassium (18.02%; 24.69%), sodium (15.57%; 21.17%), and iron (14.50%; 23.28%), respectively. The behavior of minerals during blanching is related to their solubility. Potassium, the most abundant mineral in vegetables, is extremely mobile and is easily lost by leaching during blanching because of its high solubility in water. Calcium and magnesium are generally bound to the plant tissue and are not readily lost by leaching and sometimes can even be taken up by vegetables during blanching from the processing water in areas with hard water [34]. 

### 3.3. Ascorbic Acid

**Figure 1:**
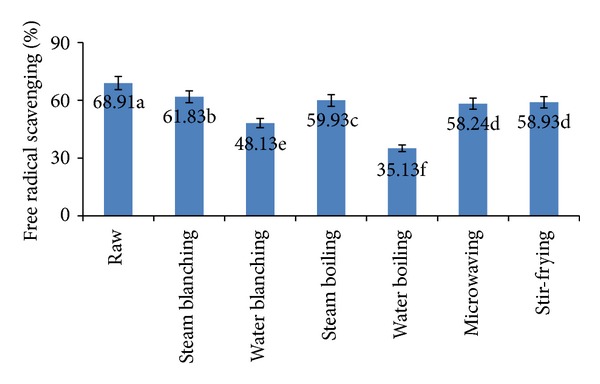
Effect of different blanching and cooking methods on vitamin C content. Results represent the means of three experiments.

Ascorbic acid is one of the most sensitive vitamins. For this reason, it is often used to evaluate the influences of food processing on vitamin contents [35]. Results in Figure 1 represent the effect of different blanching and cooking methods on ascorbic acid content of cauliflower florets. These results reveal that fresh cauliflower contained the highest level of ascorbic acid 769.23 mg/100 g (on dry weight basis). Blanching and cooking treatments caused significant reductions in ascorbic acid content ranging from 18.86% to 41.99%. The highest loss was observed for water-blanched and water-boiled cauliflower 38.69% and 41.99%, respectively. The loss of ascorbic acid can probably be ascribed to water leaching and its thermal degradation, as already reported by Lee and Kader [36]. However, steam-blanched, steam-boiled, microwaved, and stir-fried cauliflower had significantly the highest retention of ascorbic acid content. Boiling leads to high losses in ascorbic acid content levels while stewing, steaming, microwave cooking, and even pressure steaming cause only small losses [33]. Stir-frying resulted in a better retention of ascorbic acid content than cooking with a lot of water or using a microwave [37]. 

### 3.4. Total Carotenoids

Carotenoids are plant pigments that are present in human diet as microcomponents of fruits and vegetables [38]. These are a group of aliphatic-alicyclic, fat-soluble, yellow-to-red compounds responsible for red, orange, and yellow color of edible fruits and vegetables and widely distributed in nature [39].

**Figure 2:**
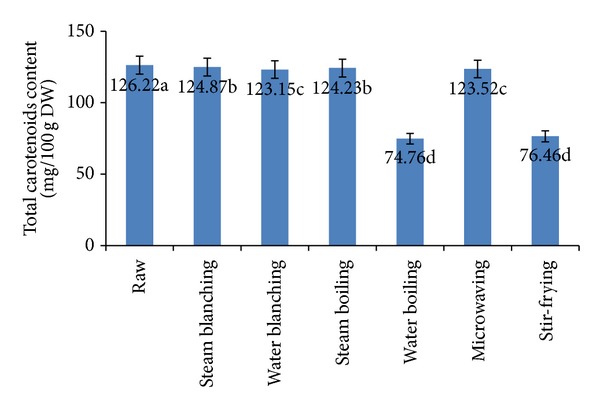
Effect of different blanching and cooking methods on total carotenoids content. Results represent the means of three experiments.

Results in Figure 2 illustrate the effect of different blanching and cooking methods on total carotenoids in cauliflower. These results reveal that fresh cauliflower contained 126.22 mg/100 g carotenoids (on dry weight basis). Water boiling and stir-frying caused significant decreases of total carotenoids, 40.77% and 39.42%, respectively. Yuan et al. [12] and Vallejo et al. [40] reported that boiling and stir-frying of broccoli resulted in significant decrease in carotenoids. In this respect, Miglio et al. [41] reported that frying of broccoli caused a 67% loss of the initial carotenoid concentration. This decrease of carotenoids of fried cauliflower may be due to the fact that carotenoids are fat-soluble compounds and readily solubilized during stir-frying and thus resulted in decrease of its contents [42]. On the other hand, there are no significant differences in the content of carotenoids of raw, steam-blanched, water-blanched, steam-boiled and microwaved cauliflower. In general, carotenoid content of foods is not altered to a great extent by common household cooking methods such as microwave cooking, steaming, and boiling, but extreme heat can result in oxidative destruction of carotenoids [43].

### 3.5. Total Phenolic Compounds

**Figure 3:**
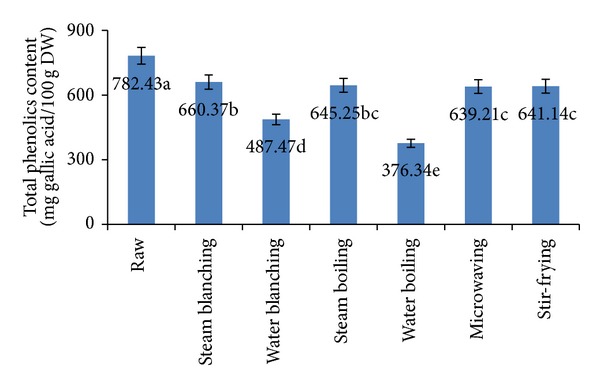
Effect of different blanching and cooking methods on total phenolics content. Results represent the means of three experiments.

Phenolic compounds (PCs) may contribute directly to the antioxidant action; therefore, it is necessary to investigate total phenolic content.  Figure 3 shows the effect of different blanching and cooking methods on total phenolic contents of cauliflower. The initial level of total phenolic in fresh cauliflower was 782.43 mg/100 g (on dry weight basis). Blanching and cooking treatments caused significant losses (15.6% to 51.9%) of total phenolic compounds. Phenolic compounds in vegetables are present in both soluble forms and combined with cell-wall complexes. Thus, increased surface area of tissues in contact with cooking water and high cooking temperatures and lengthy cooking times are all likely to have caused disruption of cell walls and breakdown of phenolic compounds [44]. The highest loss was observed for water-boiled and water-blanched cauliflower, 51.90% and 37.69%, respectively. This could be due to the breakdown of phenolics or losses (leached out) during cooking as most of the bioactive compounds are relatively unstable to heat and easily solubilized [45]. Steam-blanched, steam-boiled, stir-fried, and microwaved cauliflower had significantly the lower losses 16.6%, 17.53%, 18.05% and 18.30%, respectively. During steaming, phenolic compounds can remain in the edible part of broccoli, probably owing to the inactivation of oxidative enzymes [40]. Microwaving was better in retention of total phenolics than other cooking methods, while boiling significantly decreased the contents of total phenolics of *Boletus* mushrooms [46].

### 3.6. Total Flavonoids

**Figure 4:**
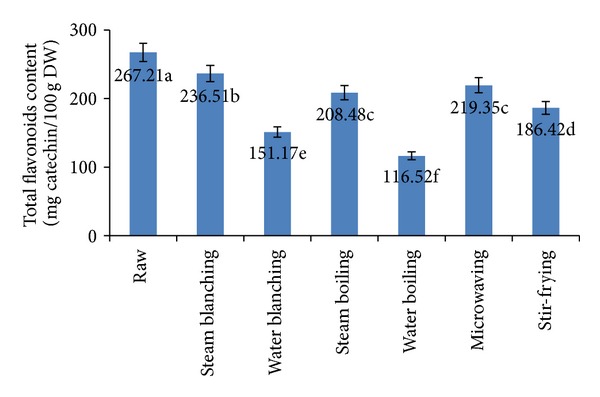
Effect of different blanching and cooking methods on total flavonoids content. Results represent the means of three experiments.

Flavonoids have a wide range of biological activities such as cellproliferation-inhibiting, apoptosis-inducing, enzyme-inhibiting, antibacterial, and antioxidant effects [47, 48]. Moreover, some findings indicate that flavonoids possess various clinical properties such as antiatherosclerotic, anti-inflammatory, antitumour, antithrombogenic, antiosteoporotic, and antiviral effects [47, 48].  Figure 4 shows the effect of different blanching and cooking methods on total flavonoids contents of cauliflower. The highest value of total flavonoids was recorded for fresh cauliflower 267.21 mg/100 g (on dry weight basis). Total flavonoids were significantly (*P* ≤ 0.05) decreased by blanching and cooking treatments. The highest reduction was noted after water boiling (56.39%) followed by water blanching (43.42%) and stir-frying (30.23%). Porter [49] reported that boiling for 5 min resulted in a 49.55% reduction in flavonoids in purple-sprouting broccoli. However, steam blanching resulted in the greatest retention of total flavonoids, followed by microwaving and then steam boiling. Conventional boiling led to a significant loss of flavonoids (66%) from fresh raw broccoli, while high-pressure boiling caused considerable leaching (47%) of caffeoylquinic acid derivatives into the cooking water. On the other hand, steaming had minimal effects, in terms of loss, on both flavonoid and hydroxycinnamoyl derivative contents [39]. Steam heating at 0.2 MPa during 40 min induces a decrease of 25% in flavonoid content of buckwheat [50]. Similar findings were reported with microwaving at 700 W during 10 min [50] and 900 W during 120 s for barley [51]. 

### 3.7. Total Antioxidant Activity

**Figure 5:**
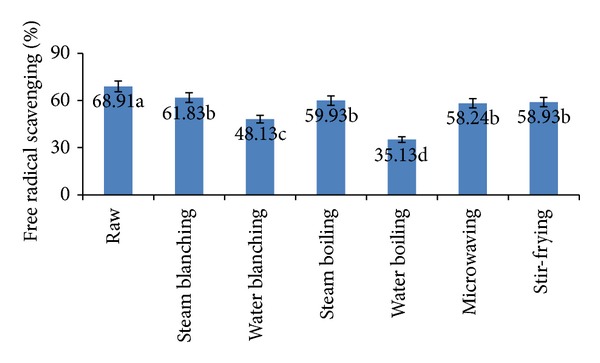
Total antioxidant activity of fresh, blanched, and cooked cauliflower. Results represent the means of three experiments.

Antioxidant activities of raw and processed cauliflower, as determined by the DPPH radical scavenging method, are shown in Figure 5. In DPPH scavenging assay, the antioxidant activity was measured by the decrease in absorbance as the DPPH radical received an electron or hydrogen radical from an antioxidant compound to become a stable diamagnetic molecule [52]. DPPH radical-scavenging activity expressed in % inhibition of the raw and processed cauliflower extracts ranged from 35.13% to 68.91%. Methanolic extract of fresh cauliflower had significantly the highest DPPH radical-scavenging activity (68.91%) followed by the extracts of steam-blanched, steam-boiled, stir-fried, and microwaved cauliflower, 61.83%, 59.15%, 58.93% and 58.24%, respectively. Kenny and O'Beirne [53] indicated that the loss of antioxidant activity was relative to the contact area between vegetables and water as well as processing time. It was clear that the contact areas in steaming and stir-frying processes were much smaller than that in boiling, so their antioxidant substances lost relatively very little [54]. Microwave heating retains the active components in the cooked tissue [55]. The activity of vegetables cooked in the microwave oven was generally higher than that of those cooked in boiling water, because microwave heating, griddling, and baking do not stimulate the release of ascorbic acid or other antioxidants from cooked tissue [56]. However, the extracts of water-boiled and water-blanched cauliflower showed the lowest scavenging activity of 35.13% and 48.13%, respectively. After boiling process, a lot of the antioxidant substances were running off into the boiling medium, which resulted in decrease of their antioxidant capacities [44]. During vegetable cooking, qualitative changes, antioxidant breakdown, and their leaching into surrounding water may influence the antioxidant activity of the vegetables [57].

### 3.8. HPLC Analysis of Phenolic Compounds of Raw, Blanched, and Cooked Cauliflower

HPLC analysis has the advantage over total phenolics content determined by the Folin-Ciocalteu method, as it provides more precise information of individual compounds. Several polyphenolic compounds were identified in fresh, blanched, and cooked cauliflower. These included gallic, pyrogallol, catechin, protocatechuic acid, catechol, chlorogenic acid, rosmarinic acid, rutin, caffeic acid, vanillic acid, quercetin, naringenin, syringic acid, coumaric acid, cinnamic, and kaempferol (Table 3). These compounds have been identified according to their retention time and the spectral characteristics of their peaks compared to those of standards as well as by spiking the sample with standards. The quantities of the identified compounds are expressed in mg/100 g DW. The predominant phenolics of raw cauliflower were protocatechuic acid (192.45), quercetin (202.4), pyrogallol (18.9), vanillic acid (11.90), coumaric acid (6.94), and kaempferol (25.91) mg/100 g DW, respectively (Table 3). Water boiling and water blanching showed the most pronounced losses of cauliflower polyphenols (−88.27 and 78.81%, resp.). Phenolic acids are dissolved in vacuoles and apoplast [58]. Cooking of vegetables determines softening and breaking of cellular components with the consequent release of these molecules into the boiling water [59]. The less negative effects on phenolic compounds were observed for steam-blanched, microwaved, steam-boiled, and stir-fried cauliflower (−57.80%, 66.08%, 69.34%, and 70.26%, resp.). Steaming and microwaving were better in retention of phenolic compounds than other cooking methods, while boiling significantly decreased the contents of these compounds of broccoli and mushrooms [40, 46]. During steaming and frying, moreover, the hydrolysis of chlorogenic acid into caffeic and quinic acids may also occur, justifying the significant increases of caffeic acid observed for both cooking methods [41]. It is worthy to mention that polyphenol losses could also be due to the covalent binding between oxidized phenols and proteins or amino acids as well as the polymerization of oxidized phenols [59].

## 4. Conclusion 

As shown in this study, blanching, boiling, microwaving, and stir-frying affect the composition, phytochemical contents, antioxidant activity, and phenolic profiles of white cauliflower. Water boiling and blanching processes caused significant losses of dry matter, protein, mineral, and phytochemical contents as well as scavenging of DPPH^∙^ radical. However, steam treatments (blanching and cooking), stir-frying, and microwaving caused slight losses, and they result in the greatest retention of nutrients and phytochemicals. 

## Figures and Tables

**Table 1:** Effect of different blanching and cooking methods on the chemical composition of cauliflower florets (g/100 g *dry weight basis*).

Component	Raw	Blanching		Boiling		Microwave cooking	Stir-frying	LSD at 0.05
		Steam	Water	Steam	Water			
Moisture	88.64^c^ ± 1.14	90.04^bc^ ± 2.07	92.62^a^ ± 1.10	91.18^ab^ ± 2.04	92.97^a^ ± 1.65	91.13^b^ ± 1.20	86.24^d^ ± 1.64	1.621
Crude protein (*N* × 6.25)	27.77^a^ ± 0.34	26.59^ab^ ± 0.85	25.75^b^ ± 0.94	26.15^ab^ ± 1.06	25.34^b^ ± 0.89	26.89^ab^ ± 0.17	25.44^b^ ± 0.30	1.282
Ether extract	2.20^b^ ± 0.14	2.09^bc^ ± 0.09	1.93^c^ ± 0.10	2.04^bc^ ± 0.09	1.92^c^ ± 0.07	2.07^bc^ ± 0.07	9.72^a^ ± 0.10	0.169
Crude fiber	11.57^b^ ± 0.41	11.56^b^ ± 0.36	11.52^b^ ± 0.54	11.54^b^ ± 0.35	12.74^a^ ± 0.38	11.54^b^ ± 0.62	11.55^b^ ± 0.21	0.746
Ash	11.03^a^ ± 0.08	9.72^ab^ ± 0.68	9.18^b^ ± 0.57	9.60^ab^ ± 0.25	7.12^c^ ± 0.30	9.65^ab^ ± 0.94	9.64^ab^ ± 1.07	1.185
Total carbohydrate*	47.43^a^ ± 1.39	50.64^a^ ± 1.99	53.72^a^ ± 2.15	51.77^a^ ± 1.76	52.88^a^ ± 1.65	50.85^a^ ± 1.85	43.65^b^ ± 3.29	3.879

Data are expressed as mean ± SD. Values given represent means of three determinations.

Means in the same row with different letters are significantly (*P* ≤ 0.05) different.

LSD: least different significantly at *P* ≤ 0.05 according to Duncan's multiple range test.

*By difference.

**Table 2:** Effect of different blanching and cooking methods on selected mineral contents of cauliflower florets (mg/100 g *dry weight basis*).

Treatment	Na	K	Ca	Mg	P	S	Zn	Mn	Fe
Raw	392^a^ ± 10.25	3657^a^ ± 12.02	480^a^ ± 8.24	450^a^ ± 9.55	329^a^ ± 22.51	720^a^ ± 9.32	25.3^a^ ± 2.01	2.15^a^ ± 0.58	26.2^a^ ± 1.96
Steam blanching	387^a^ ± 13.37	3132^c^ ± 7.42	460^ab^ ± 9.67	432^ab^ ± 8.24	308^a^ ± 31.12	712^a^ ± 5.19	24.1^a^ ± 3.18	1.95^a^ ± 0.08	24.1^a^ ± 2.83
Water blanching	331^c^ ± 6.34	2998^d^ ± 8.17	432^bc^ ± 5.82	412^b^ ± 14.27	304^a^ ± 22.01	708^a^ ± 7.02	23.5^a^ ± 4.01	1.90^a^ ± 0.41	22.4^a^ ± 1.75
Steam boiling	356^b^ ± 8.02	3120^c^ ± 5.37	454^ab^ ± 11.30	430^ab^ ± 13.29	305^a^ ± 20.11	710^a^ ± 10.34	24.6^a^ ± 1.97	1.93^a^ ± 0.37	23.6^a^ ± 0.85
Water boiling	309^d^ ± 11.78	2754^e^ ± 6.59	426^c^ ± 7.09	405^b^ ± 11.07	298^a^ ± 6.07	706^a^ ± 5.48	23.1^a^ ± 2.56	1.87^a^ ± 0.12	20.1^a^ ± 3.12
Microwaving	384^a^ ± 9.60	3241^b^ ± 13.20	462^ab^ ± 22.14	430^ab^ ± 13.06	322^a^ ± 22.16	720^a^ ± 6.77	24.2^a^ ± 3.54	2.05^a^ ± 0.64	24.4^a^ ± 5.22
Stir-frying	385^a^ ± 5.23	3238^b^ ± 22.36	458^ab^ ± 13.56	442^a^ ± 10.64	324^a^ ± 10.14	718^a^ ± 11.25	24.4^a^ ± 2.10	2.09^a^ ± 0.32	25.1^a^ ± 1.45
LSD at 0.05	16.83	20.98	21.40	20.35	36.22	14.38	5.02	0.717	4.896

Data are expressed as mean ± SD. Values given represent means of three determinations.

Means in the same row with different letters are significantly (*P* ≤ 0.05) different.

LSD: least different significantly at *P* ≤ 0.05 according to Duncan's multiple range test.

**Table 3:** HPLC analysis of phenolic compounds (mg/100 g DW) of raw and processed cauliflower.

Phenolic compounds (mg/100 g)	Raw	Steam blanching	Water blanching	Steam boiling	Water boiling	Microwaving	Stir-frying
Gallic	11.9	7.62	4.32	3.61	0.66	4.00	3.50
*Pyrogallol *	18.9	13.71	6.84	11.54	4.84	14.52	12.47
*Catechin *	1.91	0.85	ND	0.71	ND	ND	ND
*Protocatechuic *	192.4	78.78	37.23	66.10	23.04	67.41	52.38
*Catechol *	4.90	0.70	0.60	0.60	0.34	0.45	ND
Chlorogenic acid	25.83	11.36	ND	8.14	ND	12.36	10.45
*Rosmarinic acid *	1.73	ND	ND	ND	ND	ND	ND
Rutin	2.57	0.24	0.01	0.01	ND	0.12	ND
*Caffeic acid *	0.77	0.44	0.21	ND	ND	ND	1.644
*Vanillic acid *	11.90	0.031	ND	ND	ND	ND	ND
*Quercetin *	202.46	85.20	45.13	54.19	22.35	61.78	58.74
Naringenin	1.81	ND	ND	ND	ND	ND	ND
Syringic acid	1.91	ND	ND	ND	ND	ND	ND
Coumaric acid	6.94	3.69	2.94	3.48	1.54	2.62	2.10
Cinnamic acid	3.10	0.35	ND	0.25	0.24	ND	ND
Kaempferol	25.91	15.12	10.91	9.21	7.34	11.36	11.84

ND: not detected.
